# Supplementary material for: Impact of Geraniol and Geraniol Nanoemulsions on Botrytis cinerea and Effect of Geraniol on Cucumber Plants’ Metabolic Profile Analyzed by LC-QTOF-MS
Source: Plants (Basel). 2022 Sep 26;11(19):2513. doi: 10.3390/plants11192513 (PMC9571098; doi:10.3390/plants11192513)
Supplement: Supplementary file 1 [file plants-11-02513-s001.zip › plants-1914037-supplementary.pdf]

Supporting information

Article

Impact of Geraniol and Geraniol Nanoemulsions on *Botrytis cinerea* and Effect of Geraniol on Cucumber Plants' Metabolic profile analyzed by LC-QTOF-MS

Nathalie N. Kamou[1*], Natasa P. Kalogiouri[2], Panagiota Tryfon[3], Anastasia Papadopoulou[4], Katerina Karamanoli[4], Catherine Dendrinou-Samara[3] and Urania Menkissoglu-Spiroudi[1,*]

[1] Pesticide Science Laboratory, Faculty of Agriculture Forestry and Natural Environment, School of Agriculture, Aristotle University of Thessaloniki, 54124 Thessaloniki, Greece; nnkamou@gmail.com

[2] Laboratory of Analytical Chemistry, Department of Chemistry, Aristotle University of Thessaloniki, 54124 Thessaloniki, Greece; kalogiourin@chem.auth.gr

[3] Laboratory of Inorganic Chemistry, Department of Chemistry, Aristotle University of Thessaloniki, 54124 Thessaloniki, Greece; tryfon.giota@gmail.com; samkat@chem.auth.gr

[4] Laboratory of Agricultural Chemistry, Faculty of Agriculture, School of Agriculture, Forestry and Natural Environment, Aristotle University of Thessaloniki, 54124 Thessaloniki, Greece; katkar@auth.gr; anastasia17pap@hotmail.gr

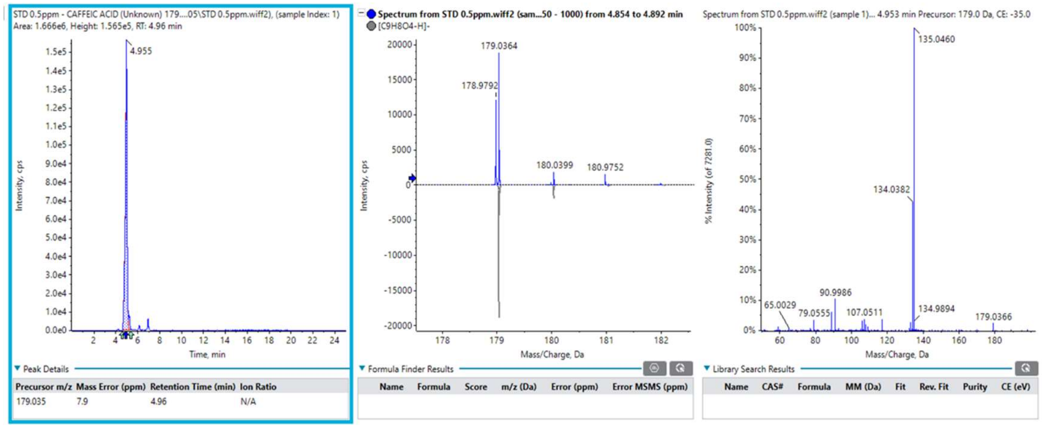

Figure S1 (a) Extracted Ion Chromatogram; (b) MS spectrum; (c) MS/MS spectrum of caffeic acid

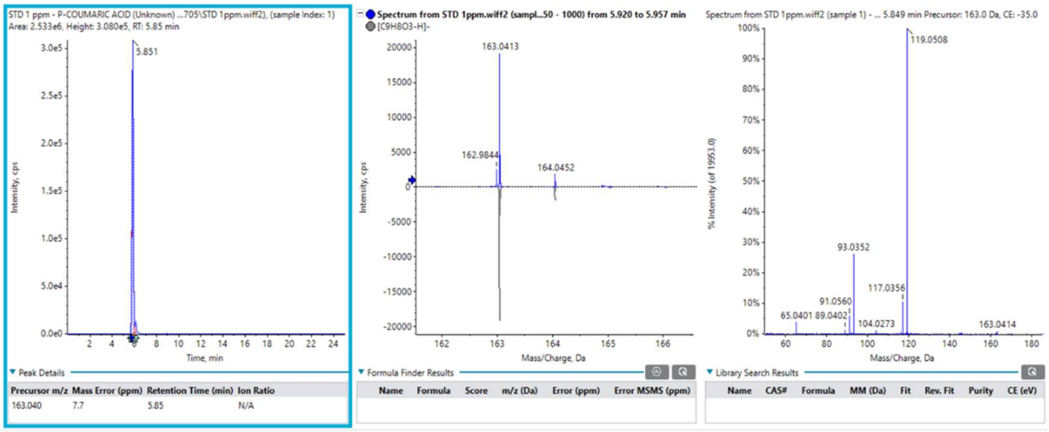

Figure S2 (a) Extracted Ion Chromatogram; (b) MS spectrum; (c) MS/MS spectrum of p-coumaric acid

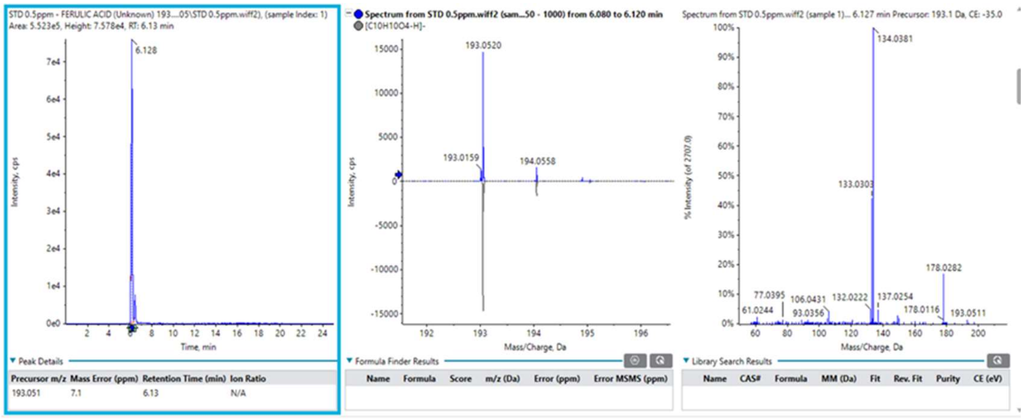

Figure S3 (a) Extracted Ion Chromatogram; (b) MS spectrum; (c) MS/MS spectrum of ferulic acid

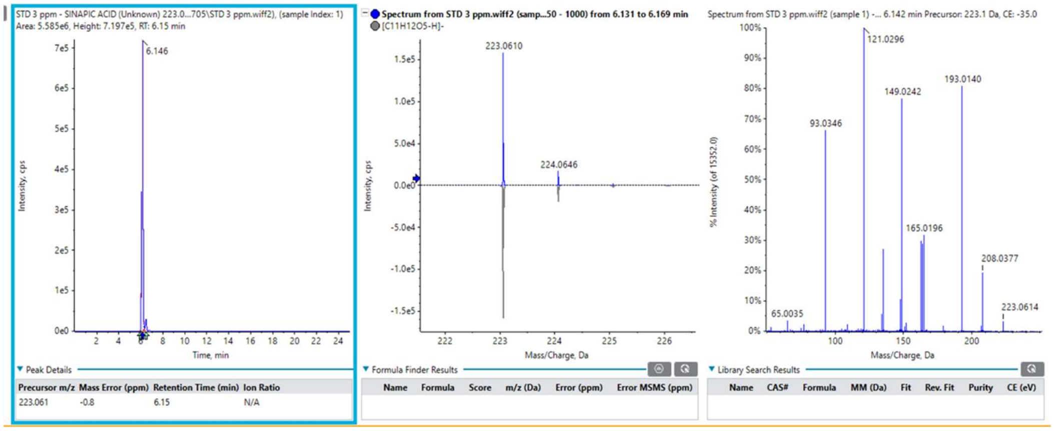

Figure S4 (a) Extracted Ion Chromatogram; (b) MS spectrum; (c) MS/MS spectrum of sinapic acid

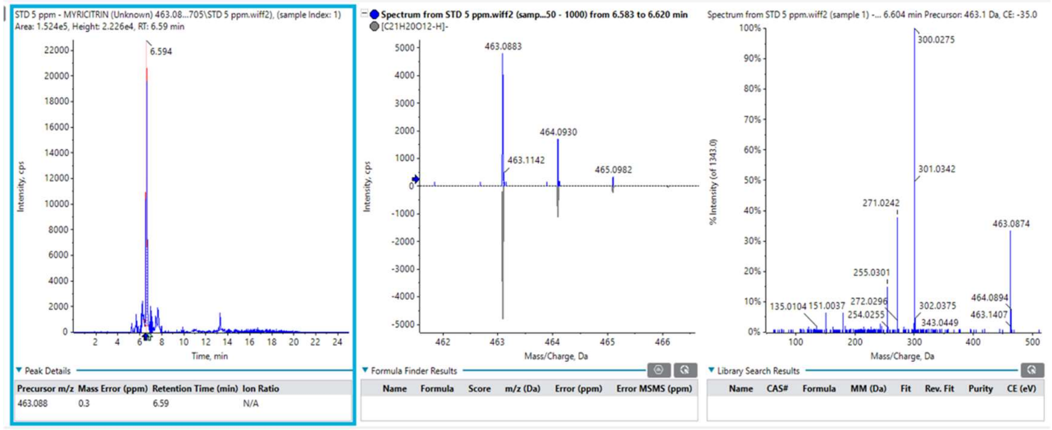

Figure S5 (a) Extracted Ion Chromatogram; (b) MS spectrum; (c) MS/MS spectrum of myricitrin

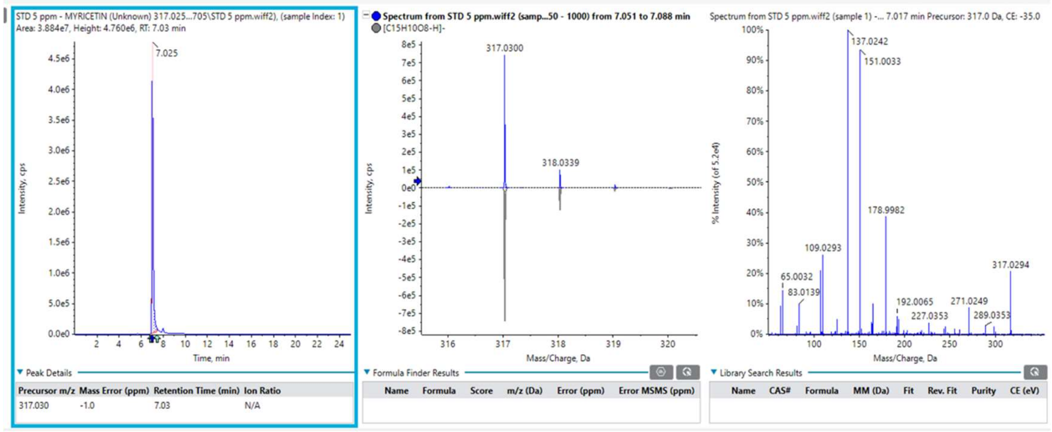

Figure S6 (a) Extracted Ion Chromatogram; (b) MS spectrum; (c) MS/MS spectrum of myricetin

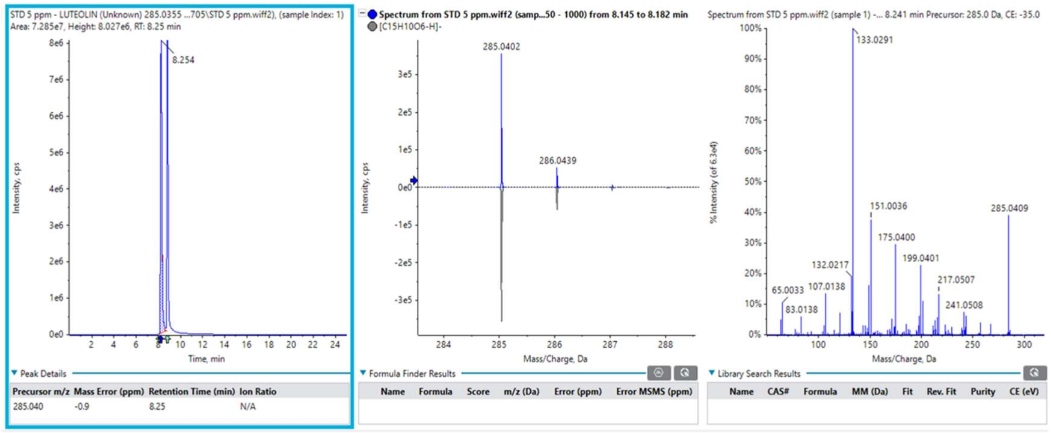

Figure S7 (a) Extracted Ion Chromatogram; (b) MS spectrum; (c) MS/MS spectrum of luteolin

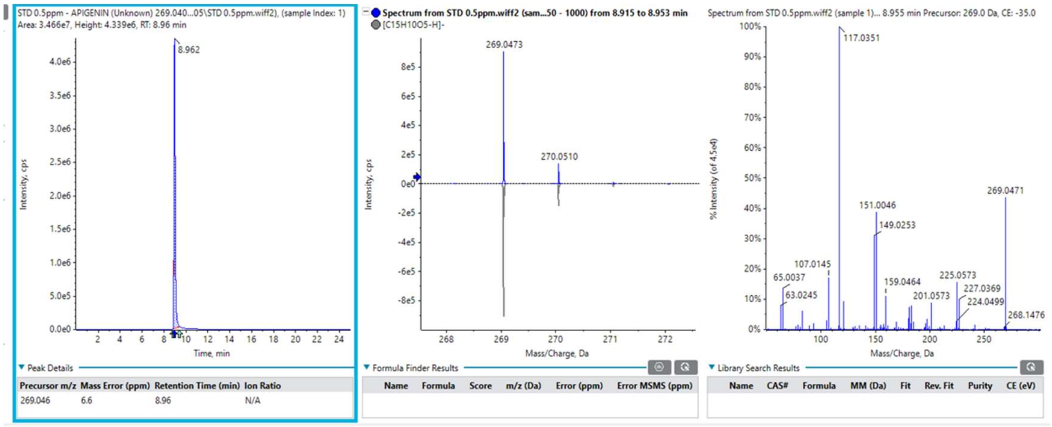

Figure S8 (a) Extracted Ion Chromatogram; (b) MS spectrum; (c) MS/MS spectrum of apigenin

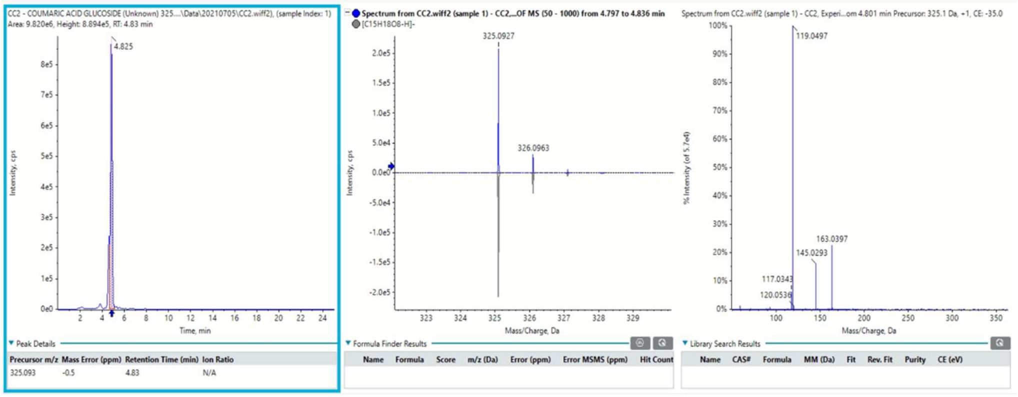

Figure S9 (a) Extracted Ion Chromatogram; (b) MS spectrum; (c) MS/MS spectrum of coumaric acid glucoside

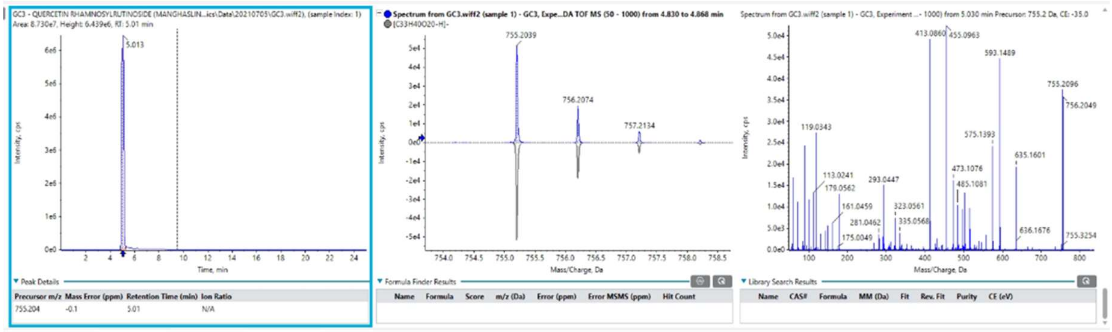

Figure S10 (a) Extracted Ion Chromatogram; (b) MS spectrum; (c) MS/MS spectrum of quercetin rhamnosyl rutinoside

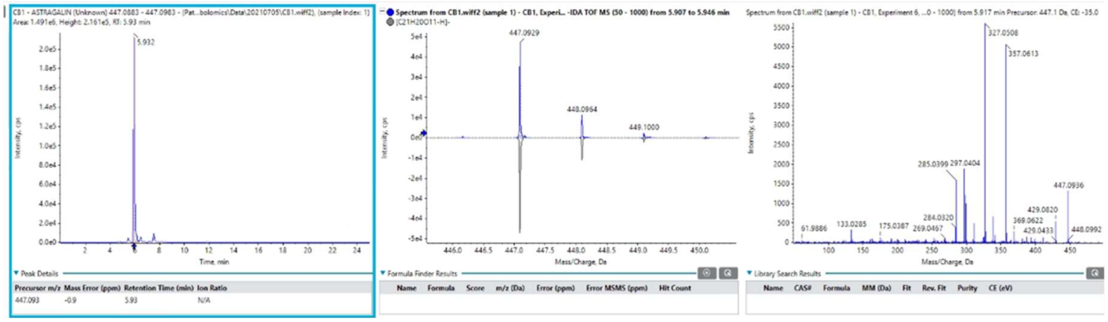

Figure S11 (a) Extracted Ion Chromatogram; (b) MS spectrum; (c) MS/MS spectrum of astragalin

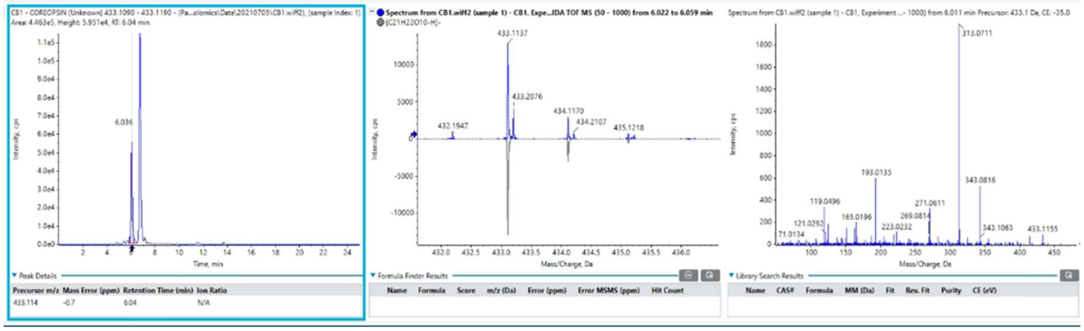

Figure S12 (a) Extracted Ion Chromatogram; (b) MS spectrum; (c) MS/MS spectrum of coreopsin

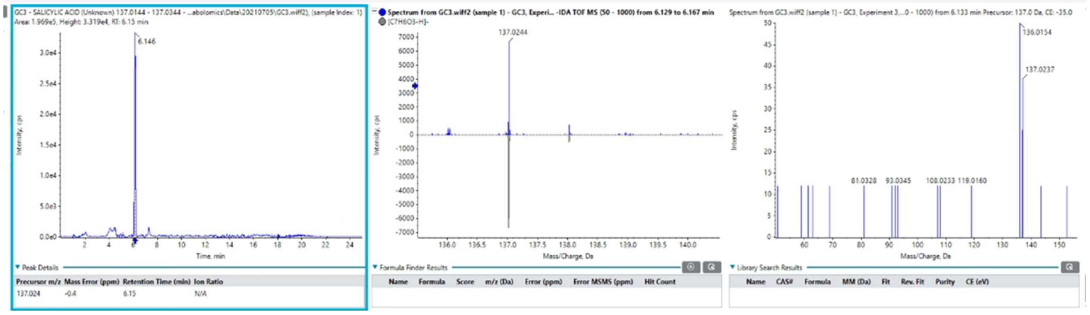

Figure S13 (a) Extracted Ion Chromatogram; (b) MS spectrum; (c) MS/MS spectrum of salicylic acid

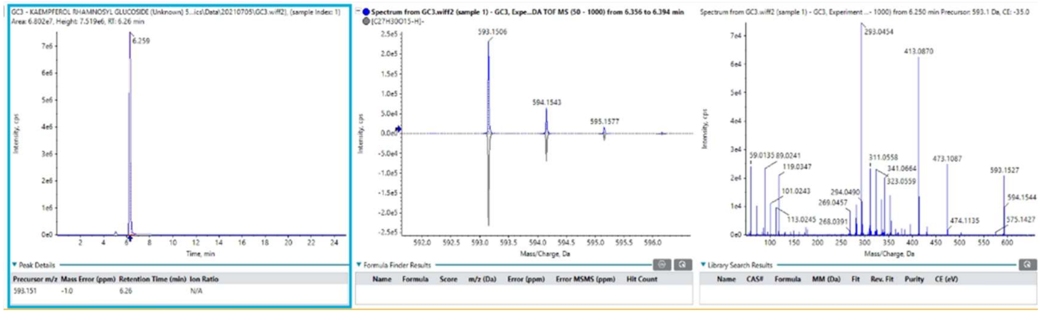

Figure S14 (a) Extracted Ion Chromatogram; (b) MS spectrum; (c) MS/MS spectrum of kaempferol rhamnosyl glucoside

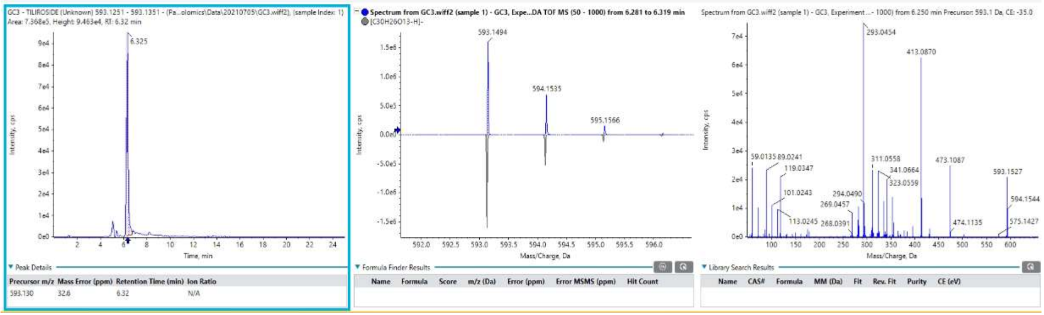

Figure S15 (a) Extracted Ion Chromatogram; (b) MS spectrum; (c) MS/MS spectrum of tiliroside

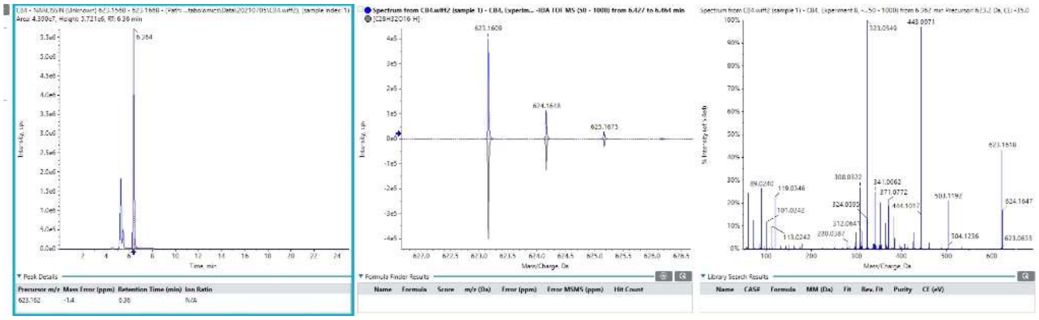

Figure S16 (a) Extracted Ion Chromatogram; (b) MS spectrum; (c) MS/MS spectrum of narcissin

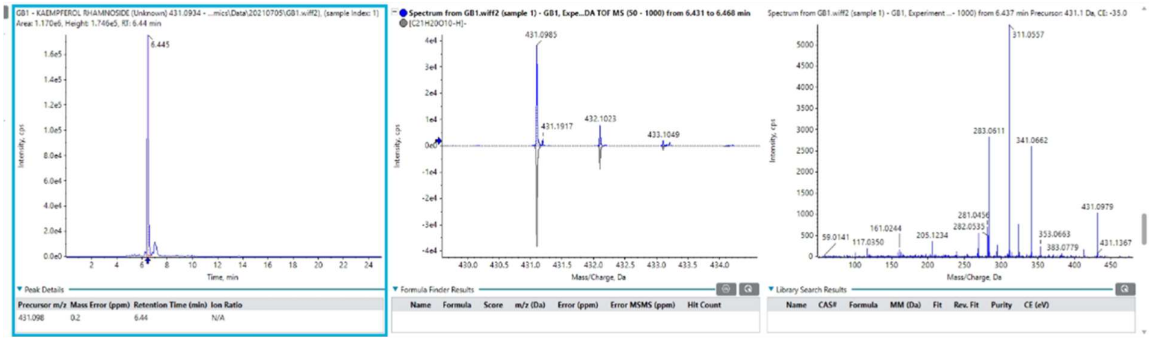

Figure S17 (a) Extracted Ion Chromatogram; (b) MS spectrum; (c) MS/MS spectrum of kaempferol rhamnoside

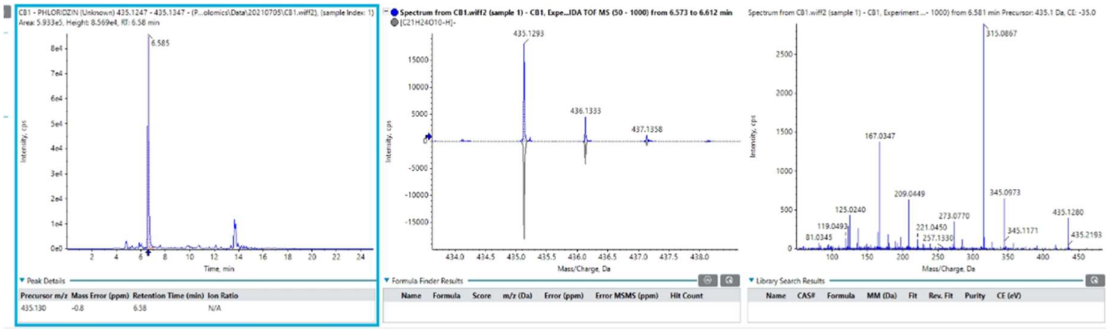

Figure S18 (a) Extracted Ion Chromatogram; (b) MS spectrum; (c) MS/MS spectrum of phloridizin

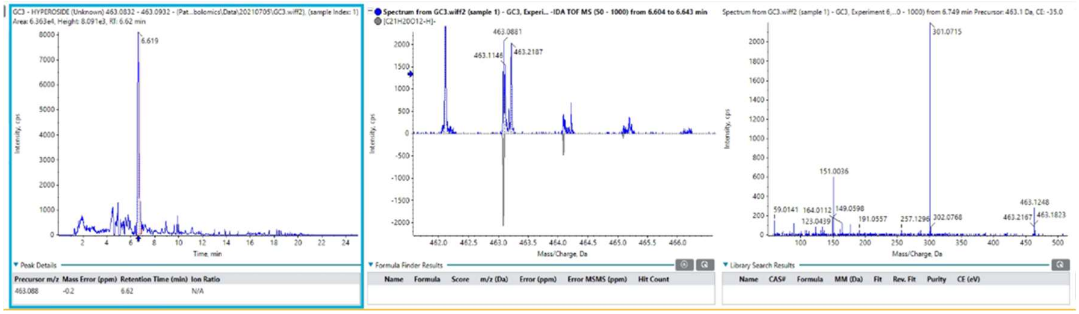

Figure S19 (a) Extracted Ion Chromatogram; (b) MS spectrum; (c) MS/MS spectrum of hyperoside

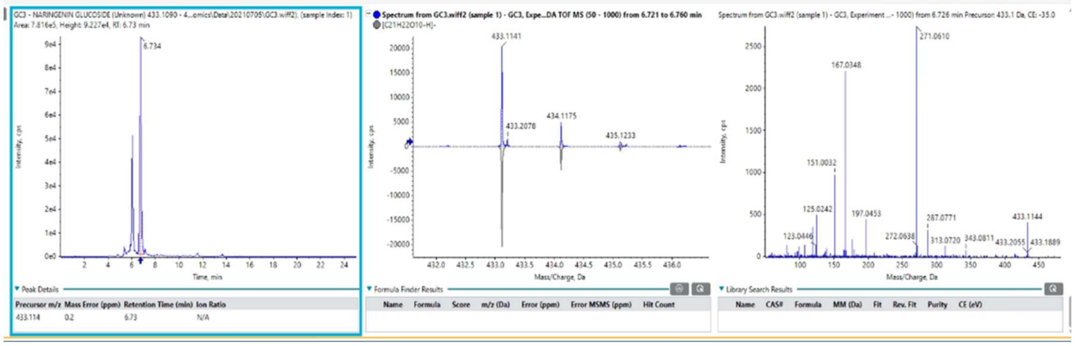

Figure S20 (a) Extracted Ion Chromatogram; (b) MS spectrum; (c) MS/MS spectrum of naringenin glucoside

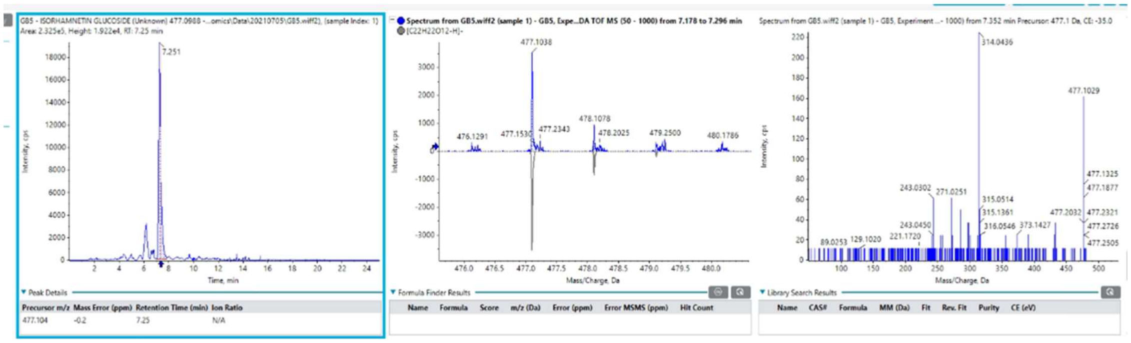

Figure S21 (a) Extracted Ion Chromatogram; (b) MS spectrum; (c) MS/MS spectrum of isorhamnetin glucoside

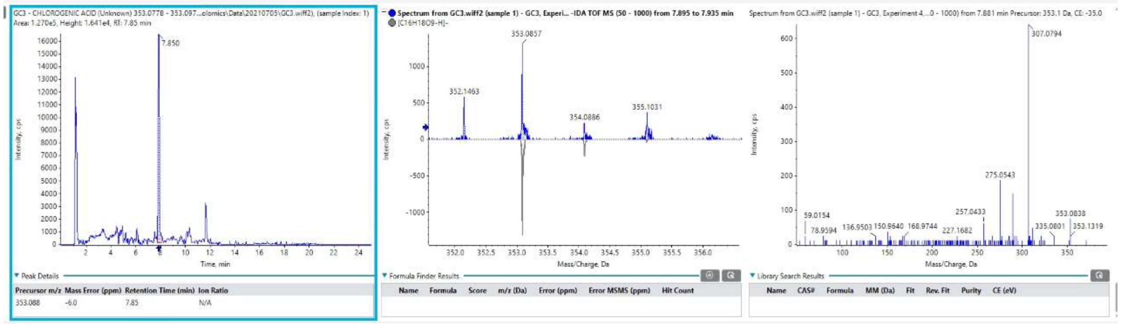

Figure S22 (a) Extracted Ion Chromatogram; (b) MS spectrum; (c) MS/MS spectrum of chlorogenic acid

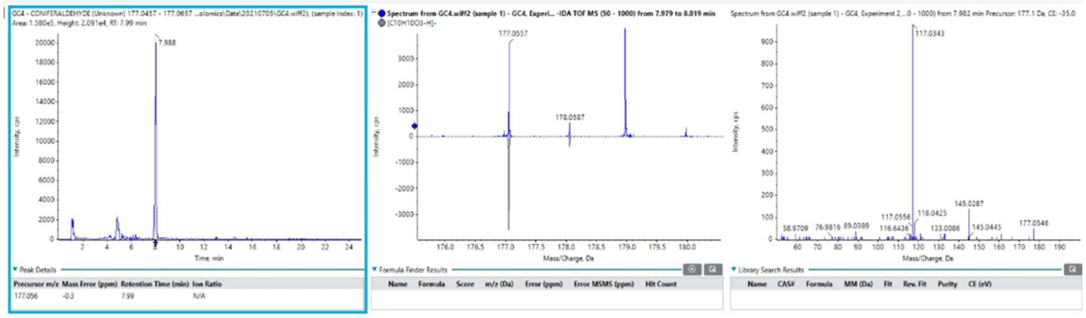

Figure S23 (a) Extracted Ion Chromatogram; (b) MS spectrum; (c) MS/MS spectrum of coniferyl aldehyde

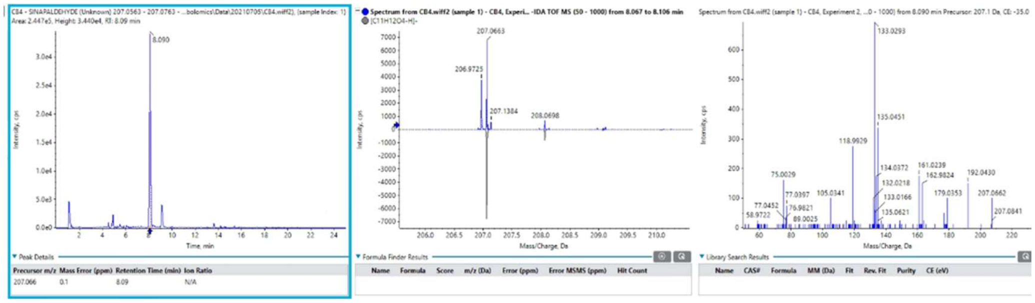

Figure S24 (a) Extracted Ion Chromatogram; (b) MS spectrum; (c) MS/MS spectrum of sinapaldehyde

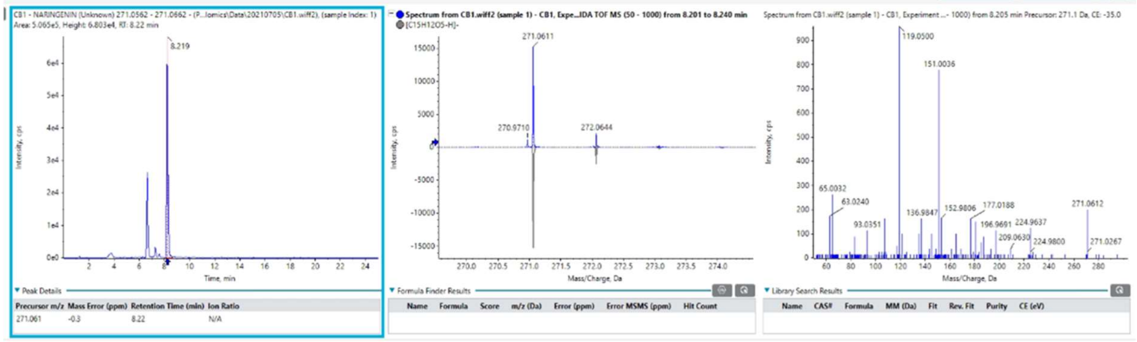

Figure S25 (a) Extracted Ion Chromatogram; (b) MS spectrum; (c) MS/MS spectrum of naringenin

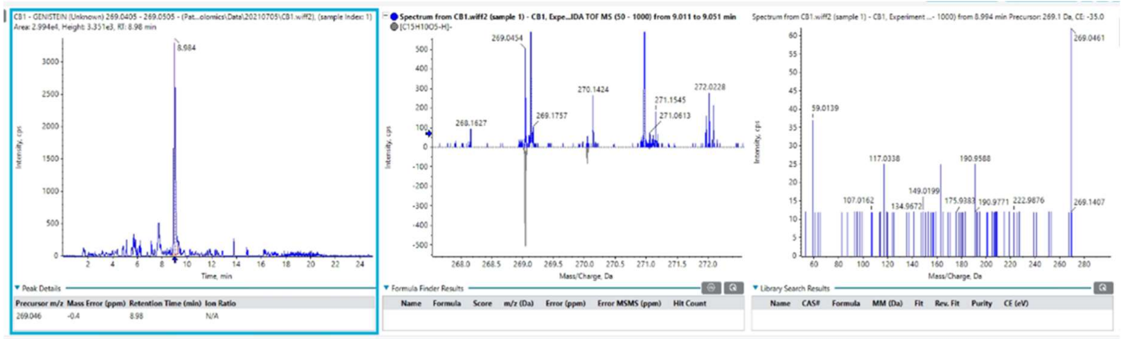

Figure S26 (a) Extracted Ion Chromatogram; (b) MS spectrum; (c) MS/MS spectrum of genistein

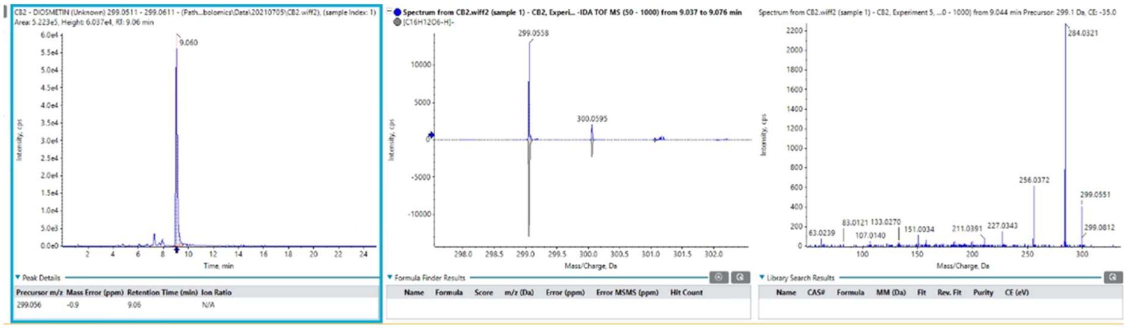

Figure S27 (a) Extracted Ion Chromatogram; (b) MS spectrum; (c) MS/MS spectrum of diosmetin
